# Effectiveness of an Empathic Chatbot in Combating Adverse Effects of Social Exclusion on Mood

**DOI:** 10.3389/fpsyg.2019.03061

**Published:** 2020-01-23

**Authors:** Mauro de Gennaro, Eva G. Krumhuber, Gale Lucas

**Affiliations:** ^1^Department of Experimental Psychology, University College London, London, United Kingdom; ^2^Institute for Creative Technologies, University of Southern California, Los Angeles, CA, United States

**Keywords:** social exclusion, empathy, mood, chatbot, virtual human

## Abstract

From past research it is well known that social exclusion has detrimental consequences for mental health. To deal with these adverse effects, socially excluded individuals frequently turn to other humans for emotional support. While chatbots can elicit social and emotional responses on the part of the human interlocutor, their effectiveness in the context of social exclusion has not been investigated. In the present study, we examined whether an empathic chatbot can serve as a buffer against the adverse effects of social ostracism. After experiencing exclusion on social media, participants were randomly assigned to either talk with an empathetic chatbot about it (e.g., “I’m sorry that this happened to you”) or a control condition where their responses were merely acknowledged (e.g., “Thank you for your feedback”). Replicating previous research, results revealed that experiences of social exclusion dampened the mood of participants. Interacting with an empathetic chatbot, however, appeared to have a mitigating impact. In particular, participants in the chatbot intervention condition reported higher mood than those in the control condition. Theoretical, methodological, and practical implications, as well as directions for future research are discussed.

## Introduction

Experiences of social exclusion, such as being isolated or excluded from a group but not necessarily ignored or explicitly disliked (Williams, 2001; Williams et al., 2005), threaten one of our most basic human needs: the desire to form strong and stable interpersonal relationships with others (Baumeister and Leary, 1995; Gonsalkorale and Williams, 2007). Being on the receiving end of social exclusion and isolation is detrimental as it can lead to a litany of negative consequences ranging from depression (Nolan et al., 2003) and low self-esteem (Leary et al., 1995) to anxiety and perceived lack of control and meaninglessness (Baumeister and Tice, 1990; Zadro et al., 2004; Stillman et al., 2009).

Unfortunately, most individuals at some point in their lives will be both sources and targets of some form of social exclusion (Williams et al., 2005). Indeed, 67% of surveyed Americans admitted giving the silent treatment to a loved one, while 75% reported having received the silent treatment from a loved one (Faulkner et al., 1997). Loneliness or feeling alone (Peplau and Perlman, 1982) are also serious problems plaguing up to a quarter of all Americans multiple times a week (Davis and Smith, 1998), with experiences of social exclusion leading to a downward spiral of further social isolation (see Lucas et al., 2010).

To date, only a few interventions have been designed to help excluded individuals recover from the adverse effects of social exclusion. Researchers have found that some interventions like emotional support animals (Aydin et al., 2012) *can* help ameliorate the negative impacts of being socially excluded. When people are excluded via social media platforms, new options open up to intervene using technology. As such, it has been shown that an online instant messaging conversation with a stranger improves self-esteem and mood after social exclusion compared to when playing a solitary computer game (Gross, 2009). In this paper, we consider a similar intervention that is more accessible: an empathetic chatbot. Specifically, we tested the possibility that an empathetic chatbot could be used to mitígate the negative impacts of exclusion.

### Agents for Mental Health

Virtual humans or agents (i.e., animated characters that allow people to interact with them in a natural way via speech or, in the case of chatbots, via text) have been designed to address various aspects of mental health and social functioning. For example, virtual agents serve as role players in mental health-related applications such as virtual reality exposure therapy (VRET). For this, therapists use agents as part of a scenario designed to evoke clinical symptoms (e.g., PTSD, social anxiety, fear of public speaking) and then guide patients in managing their emotional responses (Jarrell et al., 2006; Anderson et al., 2013; Batrinca et al., 2013). Virtual agents have also been developed to prevent such disorders and symptoms in the first place (e.g., Rizzo et al., 2012). Likewise, virtual agents can help identify disorders and symptoms by interviewing patients about their mental health (e.g., DeVault et al., 2014), and initial evidence suggests that they may be able to evoke greater openness about mental health than human clinical interviewers (Slack and Van Cura, 1968; Lucas et al., 2014; Pickard et al., 2016) or anonymous online clinical surveys (Lucas et al., 2017).

As pointed out by recent research, there is an enormous potential that chatbots hold for addressing mental health-related issues (Følstad and Brandtzaeg, 2017; Brandtzaeg and Følstad, 2018). This bourgeoning field can trace its origins back to the chatbot ELIZA (Weizenbaum, 1967) which imitated Rogerian therapy (Smestad, 2018) by rephrasing many of the statements made by the patient (e.g., if a user were to write “*I have a brother*,” ELIZA would reply “*Tell me more about your brother*”). Following ELIZA, a litany of chatbots and other applications were developed to provide self-guided mental health support for symptom relief (Tantam, 2006). A meta−analysis of 23 randomized controlled trials found that some of these self-guided applications were as effective as standard face-to-face care (Cuijpers et al., 2009). Likewise, embodied conversational agents (ECAs) can be used in cognitive-based therapy (CBT) for addressing anxiety, mood and substance use disorders (Provoost et al., 2017). Chatbots which receive text inputs from users are also beneficial as “virtual agents” in supporting self-guided mental health interventions. For example, the chatbot Woebot (Lim, 2017) guides users through CBT, helping users to significantly reduce anxiety and depression (Fitzpatrick et al., 2017).

These applications can offer help when face-to-face treatment is unavailable (Miner et al., 2016). Additionally, they may assist in overcoming the stigma around mental illness. People expect therapeutic conversational agents to be good listeners, keep secrets and honor confidentiality (Kim et al., 2018). Since chatbots do not think and cannot form their own judgments, individuals feel more comfortable confiding in them without fear of being judged (Lucas et al., 2014). These beliefs help encourage people to utilize chatbots. As such, participants commonly cite the agents’ ability to talk about embarrassing topics and listen without being judgmental (Zamora, 2017).

#### Agents for Emotional and Social Support

Similar but distinct efforts have been made to develop virtual agents and chatbots for companionship. Much of this work has focused on robotic companions for the elderly. For example, research suggests that Paro – a furry robotic toy seal- may have therapeutic effects which are comparable to live animal therapy. The robot provides companionship to the user by vividly reacting to the user’s touch using voices and gestures. Randomized control trials found that Paro reduced stress and anxiety (Petersen et al., 2017) as well as increased social interaction (Wada and Shibata, 2007) in the elderly. Virtual agents, including chatbots, also exist for companionship in older adults (Vardoulakis et al., 2012; Wanner et al., 2017), such as during hospital stays (Bickmore et al., 2015). Moreover, users sometimes form social bonds with agents (i.e., designed for fitness and health purposes) not originally intended for companionship (Bickmore et al., 2005).

Besides their potential for companionship, conversational agents have been developed to provide emotional support. When people experience negative emotions or stress, they often talk to others about their problems and seek comfort from them. Multiple studies have shown that access to support networks has significant health benefits in humans (Reblin and Uchino, 2008). For example, socio-emotional support leads to lower blood pressure (Gerin et al., 1992), reduces the chances of having a myocardial infarction (Ali et al., 2006), decreases mortality rates (Zhang et al., 2007), and helps cancer patients feel more empowered and confident (Ussher et al., 2006).

With regard to human-computer interaction, there is evidence suggesting that chatbots and virtual agents have the potential to reduce negative emotions such as stress (Prendinger et al., 2005; Huang et al., 2015), emotional distress (Klein et al., 2002), and frustration (Hone, 2006), as well as comfort users. Talking to chatbots about negative emotions or stressful experiences may also have benefits over discussing these issues with other humans. Such disclosure to chatbots can have similar emotional, relational and psychological effects as disclosing to another human (Ho et al., 2018) even though – or perhaps because – machines cannot experience emotions or judgment (Bickmore, 2003; Bickmore and Picard, 2004; Lucas et al., 2014; but see also Liu and Sundar, 2018).

Despite people’s doubt that machines can have emotional experiences, they typically respond better to agents that express emotions than those that do not (de Melo et al., 2014; Zumstein and Hundertmark, 2017). This fact can be leveraged to make emotional support agents more effective by having them display empathetic responses. In order to comfort someone in a state of grief or distress, it is known that humans employ different communicative behaviors aimed at reducing the emotional distress of another individual (Burleson and Goldsmith, 1996): one such action is empathic behavior. In the context of human-computer interaction, the empathizing agent communicates his or her understanding of the other individual’s emotional state (Reynolds, 2017). Recipients find such responses comforting, resulting in positive impacts on their well-being and health outcomes (Bickmore and Fernando, 2009).

Chatbots may then be able to use empathetic responses to support users just like humans do (Bickmore and Picard, 2005). For example, Brave et al. (2005) found that virtual agents that used empathetic responses were rated as more likeable, trustworthy, caring, and supporting compared to agents that did not employ such responses. As such, the more empathic feedback an agent provides, the more effective it is at comforting users (Bickmore and Schulman, 2007; see also Nguyen and Masthoff, 2009).

#### Social Exclusion

Interestingly, little to no work has been done to explore the possibility that virtual agents can provide effective support – *specifically* – after experiences of social exclusion. This is surprising given that social exclusion poses a particular risk because, like mental health symptoms, it can culminate over time in pervasive negative consequences. There is a large body of evidence indicating that experiences of social exclusion are associated with detrimental effects such as depression (Nolan et al., 2003), low self-esteem (Leary et al., 1995), feelings of loneliness, helplessness, frustration and jealousy (Leary, 1990; Williams, 2007), anxiety (Baumeister and Tice, 1990), lower control and belonging (Zadro et al., 2004), and reduced perceptions of life as being meaningful (Stillman et al., 2009), Moreover, being repeatedly rejected by others has been associated with attempted suicide (Williams et al., 2005) – and even with school shootings (Leary et al., 2003).

From an evolutionary perspective, it has been argued that humans developed an enhanced sensitivity to detect cues indicative of social exclusion because such state was often a death sentence to our ancestors (Pickett and Gardner, 2005). In fact, group membership enhances the species’ survival in non-human primates and animals (Silk et al., 2003), while being ostracized is associated with increased mortality (Lancaster, 1986). It is not surprising then that experiences of social exclusion are painful and distressing, leading to intense negative psychological reactions (Williams, 2007, 2009).

An extensive literature demonstrates that social exclusion causes pain and dampens mood. First, social exclusion is a painful experience which activates similar regions in the cortex as physical pain. For instance, Eisenberger et al. (2003) and Eisenberger and Lieberman (2004) collected fMRI data following experiences of social exclusion and found heightened activation of the dorsal anterior cingulate cortex (dACC), which is also activated during physical pain. Additionally, measurements from ERP, EMG, and EEG confirmed that exclusion has well developed neurobiological foundations (Kawamoto et al., 2013), and through these neurological mechanisms, social exclusion can even cause people to *feel cold* (Zhong and Leonardelli, 2008; IJzerman et al., 2012). Furthermore, social exclusion not only hurts when it comes from loved ones or in-group members, it is also distressing and painful when the person is excluded by out-group members (Smith and Williams, 2004). More importantly for the current work, research has shown that social exclusion regardless of the source can negatively affect mood (e.g., Gerber and Wheeler, 2009; Lustenberger and Jagacinski, 2010) by lowering positive affect and increasing negative affect (see Williams, 2007 for a review).

Until now, only a handful of studies have explored approaches to help excluded individuals recover from the adverse effects of social exclusion. While some interventions (e.g., emotional support animals or online instant messaging with a stranger, Gross, 2009; Aydin et al., 2012) may help improve self-esteem and mood, no controlled studies yet exist that investigate whether virtual agents could serve as a buffer against the detrimental effects of social exclusion. In this paper, we begin to explore this possibility by testing the effectiveness of an agent (i.e., an empathetic chatbot) in helping people recover from a particular detrimental effect of social exclusion: dampened mood.

#### Present Research

We begin the foray into this area of research with an empathetic chatbot designed to restore mood after social exclusion. Virtual agents may have the potential to help address this problem. As described above, when chatbots act in the role of humans, they can effectively provide emotional support. This possibility relies on our basic willingness to treat agents like humans. In Media Equation Theory, Nass and colleagues posit that people will respond fundamentally to media (e.g., fictional characters, cartoon depictions, virtual humans) as they would to humans (e.g., Reeves and Nass, 1996; Nass et al., 1997; Nass and Moon, 2000; see also Waytz et al., 2010). For instance, when interacting with an advice-giving agent, users try to be as polite (Reeves and Nass, 1996) as they would with humans. However, such considerations are not afforded to other virtual objects that do not act or appear human (Brave and Nass, 2007; Epley et al., 2007; see also Nguyen and Masthoff, 2009; Khashe et al., 2017). For example, people are more likely to cooperate with a conversational agent that has a human-like face rather than, for instance, an animal face (Friedman, 1997; Parise et al., 1999). Furthermore, it has been found that chatbots with more humanlike appearance make conversations feel more natural (Sproull et al., 1996), facilitate building rapport and social connection (Smestad, 2018), as well as increase perceptions of trustworthiness, familiarity, and intelligence (Terada et al., 2015; Meyer et al., 2016) besides being rated more positively (Baylor, 2011).

Importantly for this work, there is also some suggestion that virtual agents might be capable of addressing a person’s need to belong like humans do (Krämer et al., 2012). For example, Krämer et al. (2018) demonstrated that people feel socially satiated after interacting with a virtual agent, akin to when reading a message from a loved one (Gardner et al., 2005). Because they are “real” enough to many of us psychologically, empathetic virtual agents and chatbots may often provide emotional support with greater psychological safety (Kahn, 1990). Indeed, when people are worried about being judged, some evidence suggests that they are more comfortable interacting with an agent than a person (Pickard et al., 2016). This occurs during clinical interviews about their mental health (Slack and Van Cura, 1968; Lucas et al., 2014), but also when interviewed about their personal financial situation (Mell et al., 2017) or even during negotiations (Gratch et al., 2016). As such, the possibility exists that interactions with empathetic chatbots may be rendered safer than those with their human counterparts.

We posited that this mechanism can be adopted to help comfort participants after an experimentally induced experience of social exclusion. Many paradigms exist for inducing social exclusion, including online versions of the ball tossing game (Cyberball; Williams et al., 2000; Hartgerink et al., 2015), an online chatroom (Molden et al., 2009), and more recently a social media based social exclusion paradigm (Ostracism Online; Wolf et al., 2015). The latter simulates a social media platform such as Facebook, in which participants are excluded by receiving far fewer “likes” that other users.

Using the Ostracism Online paradigm, participants in the present work were exposed to an experience of social exclusion. Expecting to replicate prior work (e.g., Gerber and Wheeler, 2009; Lustenberger and Jagacinski, 2010; see Williams, 2007 for a review), we predicted that:

H_1_: Being excluded on the social media platform will negatively impact participants’ mood, decreasing positive emotion and increasing negative emotion from pre- to post-exclusion.

Previous studies have established that some interventions can help excluded individuals recover from the adverse effects of social exclusion (Gross, 2009; Aydin et al., 2012). Here, we explore the possibility that an empathetic chatbot can buffer against the negative effects of social exclusion, particularly dampened mood. Therefore, our second and primary prediction is that:

H_2_: After being socially excluded, experiencing a conversation with an empathic chatbot will result in better mood than a comparable control experience.

To test this possibility, we created a chatbot called “Rose” to comfort participants who had just experienced social ostracism. Informed by previous research in affective computing (Picard, 2000), Rose provided empathetic responses to help them recover from the experience. To isolate the effect of such an empathetic chatbot, participants either talked about their social exclusion experiences with Rose which responded empathically (e.g., “I’m sorry that this happened to you”) or took part in a control condition where their responses about the experience of social exclusion were merely acknowledged (e.g., “Thank you for your feedback”). The control condition ruled out the possibility that any differences in mood were simply due to participants disclosing personal feelings and then letting go of them. Instead, following Chaudoir and Fisher (2010), we wanted to demonstrate that the effect of talking is caused by receiving social support from the empathetic chatbot rather than through alleviating inhibition, where disclosure is beneficial merely because it allows people to express pent up emotions and thoughts (e.g., Lepore, 1997; Pennebaker, 1997).

## Materials and Methods

### Participants and Design

One hundred and thirty-three participants were recruited via a department subject pool, and took part in the experiment in exchange for monetary payment. Due to technical issues, data from five participants were not usable, leaving a total of 128 (94 women; *M*_age_ = 24.12, *SD* = 5.91). A power analysis using G^∗^Power (Faul et al., 2007) indicated that this sample size enabled approximately 80% power to detect a medium-sized effect of condition (Cohen’s *d* = 0.50, α = 0.05, two-tailed). The study received ethical approval from the Department of Experimental Psychology, University College London.

### Procedure

Informed consent was obtained prior to participation. Participants arrived individually at the laboratory, and were seated in front of a computer workstation running the Windows operating system with a 21^″^ screen displaying Mozilla Firefox web browser. After providing demographic information, they were told that the study was on social media profiles.

To test H_1_, participants were first exposed to the Ostracism Online paradigm (Wolf et al., 2015). It consists of a web-based ostracism task that has the appearance of a social media platform like Facebook and uses “likes” to either socially exclude or include participants. For the purpose of this experiment, participants were led to believe that they were going to interact with other students from the university with whom they would be connected via the internet. In reality, scripts were used to automate the experience on the platform. Minor changes were made to the original task by reducing the number of scripted “participants” from 11 to 6 to facilitate group cohesion (Bales and Borgatta, 1966; Tulin et al., 2018), and by altering some of the social media profiles to better resemble London university students.

**FIGURE 1:**
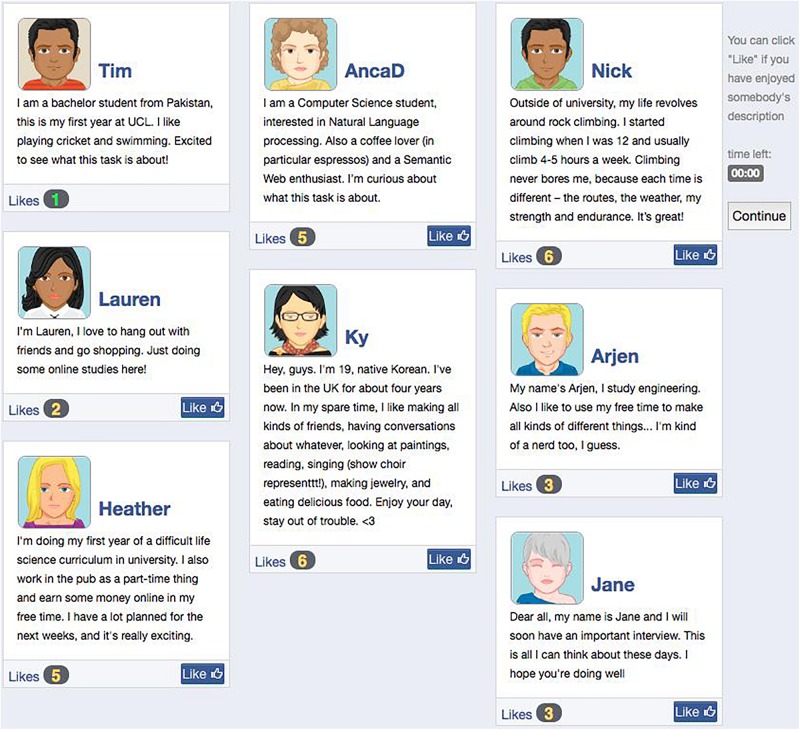
Social media exclusion induction with profile descriptions and number of “likes” for all group members. The participant’s own biography always appeared on the top left corner, receiving one “like.”

At the beginning of the task, participants were asked to create a social media profile, i.e., choose an avatar for themselves (from among 82 options), provide a name, and write a description to introduce themselves to the other people in the group. Upon completion of the profile, they were sent to a “group page” that displayed their profile alongside those of six other students (see Figure 1). Participants could “like” another profile using a button that appeared under it. Whenever participants received a “like,” a pop-up message notified them, indicating the name of the member who had liked their description (e.g., “Nick liked your post”). The number of “likes” was tallied at the bottom of each member’s profile, which was incremented with each new “like.” To induce feelings of social exclusion, the participant’s profile received only one “like” while the other profiles received on average four “likes.”

### Experimental Conditions

**FIGURE 2:**
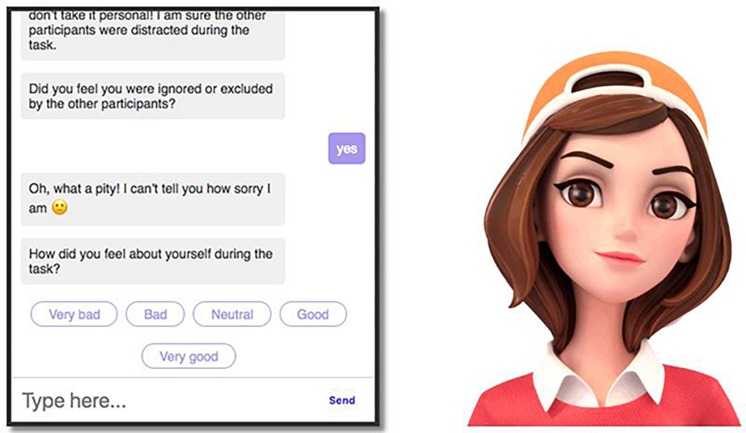
Illustration of the chat window with the empathic chatbot “Rose.”

To test H_2_, participants were then randomly assigned to one of two conditions (chatbot vs. control) in our between-subjects design. Specifically, they experienced either the empathic chatbot intervention (*n* = 64) or the control questionnaire (*n* = 64). In the chatbot intervention condition, participants interacted with a web-based embodied conversational agent named “Rose.” Rose was created using the open-source platform Botkit, and was displayed as a female agent on the right side of the chat window using the open-source library GIF-Talkr (see Figure 2). We chose a human form based on prior work showing chatbots that have a virtual body and look human-like tend to be more effective than text alone (Koda and Maes, 1996; André et al., 1998; Khashe et al., 2017), particularly females (Kavakli et al., 2012; Khashe et al., 2017).

The user interface guided users step-by-step through the chatbot conversation (Zamora, 2017). Following the use of text- and menu-based interfaces on standard social media platforms (e.g., Facebook, 2019), participants conversed with the chatbot mainly via multiple-choice menus (Bickmore and Schulman, 2007; Nguyen and Masthoff, 2009), which were updated in succession depending on the current conversation topic (Bickmore and Picard, 2005), and only occasionally using the text interface. Natural language interface was not used because of the risk for conversational errors due to poor speech recognition. Also, such errors have been shown to reduce trust and acceptance of agents (Blascovich and McCall, 2013; Wang et al., 2013; Lucas et al., 2018a), with chatbots attempting to use an entirely natural language interface being prone for failure (Luger and Sellen, 2016; see also Harnad, 1990). While multiple-choice menus were chosen over speech recognition, *agent* speech has been shown to increase trust and perceived humanness (Khashe et al., 2017; Burri, 2018; Schroeder and Schroeder, 2018). Accordingly, the chatbot used voice to interact with participants; a HTML5 Web Speech API enabled text-to-speech (TTS). Following Bhakta et al. (2014), a human-like voice (i.e., Microsoft Zira Desktop’s English voices) was selected, and the agent’s mouth movements were synchronized to speech produced by this TTS. To create a natural appearance the agent also blinked every 3–8 s, with the agent’s responses being slightly delayed to simulate a natural flow of conversation (e.g., Zumstein and Hundertmark, 2017).

The intervention started with an informal conversation in which Rose engaged in small talk about herself to create a sense of rapport and likability (Morkes et al., 1999): “Hi! My name is Rosalind, but people typically call me Rose. I am an artificial intelligence chatbot who enjoys talking to people. I can also sing and dance, but that’s probably not what you want to see right now, believe me! Anyway, I always appreciate when people try to think of me as someone with human traits, emotions, and intentions. It feels so much better to be seen as a living being.” This kind of social exchange is often used as an “icebreaker” in education and organizational fields (Clear and Daniels, 2001; Risvik, 2014; Mitchell and Shepard, 2015) to build trust and rapport, which are essential for the creation of a social relationship that makes participants feel comfortable before discussing sensitive topics (Dunbar, 1998; Bickmore and Cassell, 2000). Also, this approach has been used successfully in past studies (Inaguma et al., 2016; Lucas et al., 2018a, b). The chatbot then asked a series of questions about the social media task the participant had just completed. Throughout the conversation, empathy was expressed whenever the participant reported negative feelings. For example, when someone indicated feeling excluded or ignored (Question 7), the chatbot replied: “Oh, what a pity! I can’t tell you how sorry I am.” Similarly, if the participant stated that other members had not liked their social media biography (Question 6), the chatbot’s answer was: “I’m sorry that this happened to you, but don’t take it personal! I’m sure the other participants were distracted during the task.” When participants indicated that they had not enjoyed reading the social media biographies (Question 4), the chatbot replied: “Oh, I’m sorry to hear that:(But thank you for your honesty!” Other statements were: “Thanks for telling me. BTW, I was just curious, I wasn’t trying to test your memory;-)” (Question 2); “Oh, I’m sorry to hear that. I hope this conversation lifts your spirits” (Question 8); “It’s fine, I was being curious, I’m sure you chose a unique avatar!” (Question 3); “Oh, I’ll report it to the developers, thanks!” (Question 5). For some responses, emojis that matched the sentiment of the verbal statement were included as an additional way of providing emotional support (Smestad, 2018). In order to avoid false expectations about its capabilities (DiSalvo et al., 2002; Mori et al., 2012), the agent never explicitly used a human backstory or persona (Piccolo et al., 2018).

In contrast to the chatbot intervention condition, participants in the control condition completed an interactive questionnaire where they merely received acknowledgment that their responses were received. The number and type of questions being raised within the interface were identical to the chatbot condition. However, the conversational empathic agent was not present to provide support or comfort. For example, if a participant indicated that s/he felt ignored or excluded by the other members in the social media task (Question 7), a message like “Thank you for your feedback” was displayed to merely acknowledge receipt of their response. Similarly, if the participant stated that other members had not liked their social media biography (Question 6), the response was “Thank you for letting us know.” Other statements were: “Thanks. This question was just for statistics” (Question 2); “Thank you for your honesty” (Question 4); “We will report this to the developers, thanks” (Question 5).

### Attention and Induction Check Items

Participants then completed a number of measures, attention and induction checks, followed by dependent (i.e., mood) measures. In both conditions, participants responded to eight questions (adapted from Wolf et al., 2015) about the social media task (see Table 1). Three questions served as an attention check to ensure that participants were appropriately engaged in the task. Another two questions assessed satisfaction with the platform. The final two questions served as an “induction check” for feelings of exclusion, as we sought to confirm that the Ostracism Online paradigm was successful in inducing feelings of exclusion.

**TABLE 1:** Response frequencies for the eight questions about the social media task.

1. How many participants were connected to the social media platform?	**2**	**3**	**4**	**More than 4**	
	
	0.78% (*N* = 1)	0.78% (*N* = 1)	0.78% (*N* = 1)	97.66% (*N* = 125)	
	
2. Do you remember how many participants had female names?	**Unsure**	**2**	**3**	**4**	**More than 4**
	
	10.16% (*N* = 13)	5.47% (*N* = 7)	32.03% (*N* = 41)	32.81% (*N* = 42)	19.53% (*N* = 25)
	
3. Did any of the participants choose the same avatar as you did?	**Yes**	**No**	**Unsure**		
	
	1.56% (*N* = 2)	93.75% (*N* = 120)	4.69% (*N* = 6)		
	
4. Have you enjoyed reading the social media biographies?	**Yes**	**No**			
	
	82.81% (*N* = 106)	17.19% (*N* = 22)			
	
5. Were you able to “like” other people’s posts and see their and your “likes”?	**Yes**	**No**			
	
	96.09% (*N* = 123)	3.91% (*N* = 5)			
	
6. Do you think the other participants liked your description?	**Yes**	**No**			
	
	30.47% (*N* = 39)	69.53% (*N* = 89)			
	
7. Did you feel you were ignored or excluded by the other participants?	**Not at all**	**Slightly Excluded**	**Excluded**	**Extremely Excluded**	
	
	26.56% (*N* = 34)	56.25% (*N* = 72)	15.63% (*N* = 20)	1.56% (*N* = 2)	
	
8. How did you feel about yourself during the task?	**Very Bad**	**Bad**	**Neutral**	**Good**	**Very Good**
	
	0.78% (*N* = 1)	12.5% (*N* = 16)	57.81% (*N* = 74)	19.53% (*N* = 25)	9.38% (*N* = 12)

As shown in Table 1, the overwhelming majority of participants passed the two most obvious attention checks by correctly answering Question 1 (“More than 4”) and Question 3 (“No”). While participants were less accurate for the most challenging attention check, Question 2 (the number of participants with female names, with correct answer of “4”), given the high levels of accuracy on the other two attention checks, we take this as sufficient confirmation that participants paid attention during the experiment. On the subsequent two questions, users reported being generally satisfied with the social media platform, indicating that there were no major problems with the interface. Importantly, the Ostracism Online paradigm appeared to be effective in creating an experience of social exclusion. Most participants were aware that others did not like their profile description and the majority of participants felt at least slightly excluded. Instead of the typical skewed distribution of positive self-perception (e.g., Baumeister et al., 1989), participants showed after the ostracism task a more standard distribution for self-feelings in our study.

### Measures

A different mood measure was used to assess each hypothesis. For both measures, short instruments were selected because shorter mood scales have been shown to be more sensitive to the effect of social exclusion (Gerber and Wheeler, 2009). To test the effect of social exclusion on mood (H_1_), mood was measured before and after the social exclusion task using the 10-item version of the Positive and Negative Affect Scale (I-PANAS-SF; Thompson, 2007). Items assessed both positive affect (alert, inspired, determined, attentive, and active) and negative affect (upset, hostile, ashamed, nervous, and afraid), and were rated on a five-point Likert scale (1 = *strongly disagree*, 5 = *strongly agree*). Composite scores for positive and negative affect were both internally consistent (α = 0.84 and α = 0.80, respectively).

To test the moderating effect of the chatbot intervention (H_2_), participants were asked to also report their mood after the chatbot (or control) experience. In order to prevent participants from noticing that the scale was the same, and thus allow demand characteristics to influence responses, final mood was assessed using the question “How do you feel at this particular moment?” (1 = *extremely negative*, 7 = *extremely positive*). At the end of the study, participants were debriefed, thanked and compensated for their time. Prior to revealing the purpose of the experiment during the debriefing, no participants indicated suspicions about any aspects of the study.

## Results

To test the effect of social exclusion on mood (H_1_), we compared participants’ affect ratings before and after the Ostracism Online experience. Composite scores of the I-PANAS-SF were submitted to two paired-samples *t* tests with positive or negative mood as the dependent measure. After being socially excluded, positive affect was reduced (*M*_pre_ = 16.80, *SD* = 3.45 vs. *M*_*post*_ = 13.97, *SD* = 4.46), *t*(127) = 9.08, *p* < 0.001, Cohen’s *d* = 0.80, 95% CI [0.46,0.96], whereas negative affect increased (*M*_pre_ = 6.89, *SD* = 2.63 vs. *M*_*post*_ = 8.33, *SD* = 3.42), *t*(127) = -4.70, *p* < 0.001, Cohen’s *d* = 0.42, 95% CI [0.22,0.72]. Interpreting the confidence intervals around the effect sizes, with 95% confidence, the effect is small to large for the increase in negative affect and medium to large for the decrease in positive affect. The same results were obtained when submitting the data to a 2 (time: pre vs. post) x 2 (valence: positive vs. negative) repeated-measures ANOVA, showing significant main effects of time, *F*(1,127) = 11.64, *p* = 0.001, η_*p*_^2^ = 0.08, and valence, *F*(1,127) = 355.37, *p* < 0.001, η_*p*_^2^ = 0.74, as well as their interaction, *F*(1,127) = 85.13, *p* < 0.001, η_*p*_^2^ = 0.40. None of the above effects was moderated by condition (chatbot vs. control), *F*s < 1.03, *p*s > 0.31, indicating that mood changed as a function of the Ostracism Online experience similarly across both conditions.

To test the effect of the chatbot intervention (H_2_), an independent samples *t*-test was conducted on mood after the intervention (or control) experience. In line with predictions, a main effect of condition occurred, *t*(126) = 2.08, *p* = 0.040, Cohen’s *d* = 0.37, 95% CI [0.02,0.72]; interpreting the confidence interval around the effect size, with 95% confidence, the effect of condition is non-negative to large. After being socially excluded, those who interacted with the chatbot had significantly more positive mood (*M* = 3.77, *SD* = 0.89) compared to participants in the control condition (*M* = 3.45, *SD* = 0.82). When dropping data from participants who responded feeling “not at all” socially excluded (Question 7), the results remained the same, *M*_chatbot_ = 3.62, *SD* = 0.79, *M*_control_ = 3.30, *SD* = 0.72, *t*(92) = 2.04, *p* = 0.044, Cohen’s *d* = 0.42, 95% CI [0.13,0.71]; interpreting the confidence interval around the effect size, with 95% confidence, the effect is small to large. When considering feelings of social exclusion (Question 7) as a covariate in an ANCOVA, the main effect of condition remained significant, *M*_chatbot_ = 3.77, *SD* = 0.89, *M*_control_ = 3.45, *SD* = 0.82, *F*(1,125) = 4.72, *p* = 0.032, η_*p*_^2^ = 0.04. We are therefore confident that the results are stable, even in the context of those participants who failed the induction check for feelings of exclusion.

## Discussion

In this work, we provide initial evidence that a fully automated embodied empathetic agent has the potential to improve users’ mood after experiences of social exclusion. First, we expected to replicate previous findings that being excluded would have a negative impact on mood. Indeed, a comparison of the I-PANAS-SF scores before and after the social exclusion task revealed a significant increase in negative mood and a significant decline in positive mood. Although the effect of social exclusion on subsequent behavior may last longer than its effect on mood (Williams, 2007; Gerber and Wheeler, 2009), research has shown substantial immediate effects on mood, with exclusion lowering positive affect and increasing negative affect (see Williams, 2007 for a review).

Importantly, in our second hypothesis, we predicted that an emotional support chatbot that displays empathy would mitigate the negative effect on mood. As expected, the chatbot intervention helped participants to have a more positive mood (compared to the control condition) after being socially excluded. This result is in line with those of previous studies with emotional support chatbots designed for other purposes (e.g. Bickmore and Picard, 2005; Nguyen and Masthoff, 2009), and further supports the idea that chatbots that display empathy may have the potential to help humans recover more quickly after experiencing social ostracism.

A possible explanation for this finding could be offered by Media Equation Theory (Reeves and Nass, 1996), which states that humans instinctively perceive and react to computers (and other media) in much the same manner as they do with people. Despite knowing that computers are inanimate, there is evidence that they unconsciously attribute human characteristics to computers and treat them as social actors (Nass and Moon, 2000). Moreover, people often rely on heuristics or cognitive shortcuts (Tversky and Kahneman, 1974) and mindlessly apply social scripts from human-human interaction when interacting with computers (Sundar and Nass, 2000). Nass and Moon (2000) argue that we tend not to differentiate mediated experiences from non-mediated experiences and focus on the social cues provided by machines, effectively “suspending disbelief” in their humanness. Due to our social nature, we may fail to distinguish chatting with a bot from interacting with a fellow human. As such, there is reason to believe that people have a strong tendency to respond to the social and emotional cues expressed by the chatbot in a way as if they had originated from another person. For example, Liu and Sundar (2018) found evidence supporting the Media Equation Theory in the context of chatbots expressing sympathy, cognitive empathy and affective empathy. In line with this notion, sympathy or empathy coming from a chatbot could then have similar effects on the individual as in human-human interaction.

The present research tried to rule out the possibility that the observed differences in mood between the chatbot intervention and control conditions were due to participants disclosing about, and thus letting go of, the social exclusion. Given that participants in both conditions had the opportunity to get the exclusion experience “off their chest,” the control condition was equated to the intervention condition in terms of alleviating inhibition (Chaudoir and Fisher, 2010), allowing people to express pent up emotions and thoughts (e.g., Lepore, 1997; Pennebaker, 1997). We are therefore relatively confident that mood was restored through the provision of social support by the empathic chatbot rather than just letting users express themselves.

The present research makes important contributions. First and foremost, the work appears to be the first to evaluate the usefulness of chatbots in helping individuals deal with the negative effects of social exclusion. As such, it demonstrates the possibility of empathic chatbots as a supportive technology in the face of social exclusion. Additionally, by showing that empathetic chatbots have the potential to recover mood after exclusion on social media, the work contributes to both the social exclusion literature and the field of human-computer interaction. By adapting the Ostracism Online task (Wolf et al., 2015) for the purposes of the present research, we validated the paradigm in a different setting (i.e., laboratory) with university students rather than online via Mechanical Turk workers (see support for H_1_). Furthermore, it extends most past studies in human-computer interaction which used the Wizard of Oz methodology (see Dahlbäck et al., 1993) in which participants are led to believe that they are interacting with a chatbot when in fact the chatbot is being remotely controlled by a human confederate. The present study employed a fully automated empathic chatbot. Since this chatbot was created using free open source tools, it can be easily customized for future research or even be of applied use to health professionals. This makes a final contribution by affording opportunities for future research and applications.

### Limitations and Further Research

Before drawing conclusions regarding the effectiveness of empathic chatbots in assisting socially excluded individuals, it is essential to examine whether novelty effects contributed to the results. According to the innovation hypothesis, any social reaction toward chatbots is simply due to novelty which eventually disappears once the novelty wears off (Chen et al., 2016; Fryer et al., 2017). In future research, longitudinal studies could be conducted in order to rule out this possibility. Moreover, similar but sufficiently distinct mood scales could be used over the course of the experiment to allow for direct comparability in mood between the different time points. In the present research, a single item affect measure was employed at time 3 to prevent participants from indicating the same response several times. While such approach avoids potential demand effects, it did not allow us to measure direct change in affect from time 2 to 3. Alternatively, rather than relying on self-report scales, future studies might consider implicit measures of mood.

Another limitation was the limited number of participants that were recruited, and the relatively high *p*-value for the main effect of condition (H2). The small number of participants meant that only two conditions could be studied (chatbot vs. control), as a 2 (exclusion vs. inclusion) x 2 (chatbot vs. control) design would have resulted in insufficient statistical power. To address this shortcoming, future research should replicate this study with more participants, adding a social inclusion condition to the design. While the control condition was otherwise perfectly comparable to the chatbot intervention condition, it is important to note that the experimental design does manipulate both empathy and the presence of a chatbot. Thus, in this initial test we considered the “empathetic chatbot” as the intervention under investigation. Furthermore, the current study’s control condition differed from the intervention condition in terms of the presence of empathy and the chatbot itself. Accordingly, future research could further isolate “empathy” as the driving factor (controlling the mere presence of the chatbot) by employing a control condition with a chatbot that does not attempt to make participants feel better. While the resulting effect would likely be smaller (and thus require a larger sample size to achieve comparable statistical power), this design would increase internal validity.

Likewise, the observed effect may have been bolstered by the presence of a human-like face (compared to no face). For example, there is evidence that people perceive embodied chatbots that look like humans as more empathic and supportive than otherwise equivalent chatbots that are not embodied (i.e., text-only; Nguyen and Masthoff, 2009; Khashe et al., 2017). Future research could consider the role of embodiment by comparing the effectiveness of *embodied* empathetic chatbots for ameliorating negative effects of social exclusion to the effectiveness of equivalent chatbots that are not embodied.

While this research suggests that chatbots can help humans recover their mood more quickly after social exclusion, empathetic agents may reduce the willingness to seek social connection, especially for lonely individuals given that they fear social rejection (Lucas, 2010; Lucas et al., 2010). For example, work on “social snacking” demonstrates that social cues of acceptance (such as reading a message from a loved one) can temporarily satiate social needs and in turn reduce attempts to connect (Gardner et al., 2005). Accordingly, it is possible that agents that build connection using empathy and other rapport-building techniques could cue social acceptance, thereby lowering users’ willingness to reach out to others. Krämer et al. (2018) provided initial evidence for this possibility by demonstrating that, among those with activated needs to belong (i.e., lonely or socially isolated individuals), users were less willing to try to connect with other humans after interacting with a virtual agent. This occurred only if the agent displayed empathetic, rapport-building behavior. By meeting any outstanding immediate social needs, empathetic chatbots could therefore make users more socially apathetic. Over the long term, this might hamper people from fully meeting their need to belong. If empathetic chatbots draw us away from real social connection with other humans through a fleeting sense of satisfaction, there is an especially concerning risk for those who suffer from chronic loneliness, given they are already hesitant to reach out to others so as not to risk being rejected. As such, supportive social agents, which are perceived as safe because they will not negatively evaluate or reject them (Lucas et al., 2014), could be very alluring to people with chronic loneliness, social anxiety, or otherwise heightened fears of social exclusion. But those individuals, who already feel disconnected, are likely to not find their need to belong truly fulfilled by these “parasocial” interactions. Future research should thus consider these possibilities and seek to determine under what conditions -and for whom- empathetic chatbots are able to encourage attempts at social connection.

Finally, while this research suggests that chatbots can help humans recover their mood more quickly after social exclusion, this intervention would not serve as the sole remedy for the effect of social exclusion on mood and mental health. While intense interventions such as cognitive-behavioral therapy, acceptance and commitment therapy, and dialectical behavioral therapy can help people to learn how to cope with the negative feelings and reframe the rejection such that it does not produce such negative affect and adverse effects on mental health, there are also other simpler more subtle interventions that could be used -like empathic chatbots- to reduce the sting of rejection and its impact on mood. For example, results from Lucas et al. (2010) suggest that subtly priming social acceptance may be able to trigger “upward spiral” of positive reaction and mood among those faced with perceived rejection; this suggests that “even the smallest promise of social riches” can begin to ameliorate the negative impact of rejection.

## Conclusion

Adding to the literature on how to achieve social impact with chatbots, this study yields promising evidence that ECAs have the potential to provide emotional support to victims of social exclusion. Fully automated empathic chatbots that can comfort individuals have important applications in healthcare. In particular, they offer unique benefits such as the ability to instantly reach large amounts of people, being continuously available, and overcoming geographical barriers to care. Even if chatbots do not infiltrate healthcare, they may be effective at mitigating negative emotional effects such as those created by cyberbullying. In this and similar use cases and applications, chatbots can be deployed to support mood when users embark in the murky waters of the internet with its potential risks of negativity and hurt feelings. In such cases, empathetic chatbots should be used alongside other approaches to improve the mental health of individuals who are victims of cyberbullying. Finally, while the present results are preliminary and need to be viewed with caution, our study demonstrates the potential of chatbots as a supportive technology and sets a clear roadmap for future research.

## Data Availability Statement

The datasets generated for this study are available on request to the corresponding author.

## Ethics Statement

The studies involving human participants were reviewed and approved by the Department of Experimental Psychology, University College London. The patients/participants provided their written informed consent to participate in this study.

## Author Contributions

MG and EK conceived and designed the experiments and analyzed the data. MG performed the experiments. GL, EK, and MG contributed to writing and revising the manuscript and read and approved the submitted version.

## Conflict of Interest

The authors declare that the research was conducted in the absence of any commercial or financial relationships that could be construed as a potential conflict of interest.
